# Key Intrinsic Connectivity Networks for Individual Identification With Siamese Long Short-Term Memory

**DOI:** 10.3389/fnins.2021.660187

**Published:** 2021-06-18

**Authors:** Yeong-Hun Park, Seong A. Shin, Seonggyu Kim, Jong-Min Lee

**Affiliations:** ^1^Department of Biomedical Engineering, Hanyang University, Seoul, South Korea; ^2^Department of Electronic Engineering, Hanyang University, Seoul, South Korea

**Keywords:** dynamic resting-state fMRI, individual identification, long short-term memory, Siamese network, ROI-wise average pooling, individual uniqueness

## Abstract

In functional magnetic resonance imaging (fMRI) analysis, many studies have been conducted on inter-subject variability as well as intra-subject reproducibility. These studies indicate that fMRI could have unique characteristics for individuals. In this study, we hypothesized that the dynamic information during 1 min of fMRI was unique and repetitive enough for each subject, so we applied long short-term memory (LSTM) using initial time points of dynamic resting-state fMRI for individual identification. Siamese network is used to obtain robust individual identification performance without additional learning on a new dataset. In particular, by adding a new structure called region of interest–wise average pooling (RAP), individual identification performance could be improved, and key intrinsic connectivity networks (ICNs) for individual identification were also identified. The average performance of individual identification was 97.88% using the test dataset in eightfold cross-validation analysis. Through the visualization of features learned by Siamese LSTM with RAP, ICNs spanning the parietal region were observed as the key ICNs in identifying individuals. These results suggest the key ICNs in fMRI could represent individual uniqueness.

## Introduction

Functional connectivity (FC) analysis generally considers full-length blood-oxygen-level-dependent (BOLD) signal to observe specific brain activity patterns using resting-state functional magnetic resonance imaging (fMRI), usually acquired for 5–15 min. Using FC in fMRI studies, intrinsic connectivity network (ICN) has been identified as sets of temporally correlated and spatially independent brain activations. More recently, dynamic FC studies have been conducted involving short-time (30–60 s) BOLD signal patterns to observe temporal changes in ICN across groups and individuals. The main research interest in the field of fMRI has shifted from group differences to individual discrepancies using fMRI measures with the launch of the Human Connectome Project (HCP). In efforts to discover individual discrepancies, a large number of studies have successfully revealed that fMRI measures reflect individual characteristics. For example, it was observed that significant differences of FC and ICN between individuals were correlated with individual cognitive and behavioral score (Barch et al., 2013). Finn et al. (2015) also applied FC to identify individuals and predict individual clinical scores. As it has been established in previous findings that individual identification can be achieved with fMRI measures, it may indicate that individual’s fMRI data has a uniqueness that can represent an individual without changing over time. Cole et al. (2016) and Tavor et al. (2016) used FC to predict the individual task–specific brain activity. In studies by Liu et al. (2018) and Fong et al. (2019), individual task performance and behavior score could be predicted by using dynamic FC. These studies did not consider the dynamic sequence of BOLD signal because individual identification and individual score prediction were performed using the FC based on full-length BOLD signal or the variability of the FC over the scan time and short-time BOLD signal patterns (e.g., SD, frequency, and mean strength of each connectivity).

Long short-term memory (LSTM) is a recurrent neural network (RNN)–type deep-learning model that is excellent at recognizing time-series data to predict future data or to classify time-series data (Hochreiter and Schmidhuber, 1997). Unlike RNN, which consists of an input, output, and hidden layers, LSTM includes cell states to prevent the loss of initial time information of sequence data. Due to the aforementioned structural characteristics, LSTM remembers not only short-term memory but also long-term memory, which is advantageous for learning sequence data. Due to its advantages, LSTM has been implemented in numerous fMRI studies. Guclu and van Gerven (2017) used LSTM to estimate the hemodynamic response functions to sensory stimuli by capturing temporal dependencies in the task-related fMRI study. Furthermore, it was employed to classify normal and neurological disease groups in resting state fMRI studies (Sarraf and Tofighi, 2016; Dvornek et al., 2017; Mao et al., 2019; Yan et al., 2019). Chen and Hu (2018) and Wang et al. (2019) also applied LSTM and gated recurrent units (GRUs) (Cho et al., 2014) to classify individual subjects by resting-state fMRI, and observed spatiotemporal features for individual identification. However, their individual classification models have the disadvantage of being unable to guarantee the consistent performance when adding new subjects or evaluating new datasets because the model must be newly trained.

In this study, we applied Siamese networks (Bromley et al., 1994) to ensure robust performance on new datasets. In the Siamese networks, two identical networks extract features from two input images and calculate the distance between the feature vectors to evaluate whether the two input images are identical. At this point, the two identical networks share the weight. When learning is complete, the distance is close to zero for identical input image pairs, and the distance is close to one for non-identical input image pairs. The advantage of a Siamese neural network is that the model does not need to be retrained even if a new subject or dataset is added. In addition, we applied a new structure called region of interest (ROI)–wise average pooling (RAP) to enhance the individual identification performance of the model, which also allowed us to observe which ROIs of ICN are important for individual identification. Briefly speaking, the current study implemented Siamese LSTM with RAP to identify individual resting-state fMRI data without model retraining for new subjects. The Siamese LSTM with RAP calculated the distance between the features of individual short-time BOLD signals extracted from two identical LSTM. For the test, the identical gallery data (HCP resting-state fMRI data taken on day 1) was identified as the particular probe data (HCP resting-state fMRI data taken on day 2) without further learning about the test subjects. By visualization and occlusion methods, key ICNs for individual identification were identified.

## Materials and Methods

### fMRI Data and Preprocessing

The present study used HCP S900 data released in December 2015 (Van Essen et al., 2012). Resting-state fMRI data of the HCP were acquired twice (RL and LR phase encoding) on day 1 and day 2; thus, there were four sessions in total. The resting-state fMRI datasets analyzed for this study can be found in the HCP^1^. Among the HCP S900, 813 subjects with both resting-state fMRI data acquired as RL phase encoding on day 1 and day 2 (REST1_RL and REST2_RL; 461 females, age: 28.75 ± 3.7) were selected. The experiments were performed in accordance with relevant guidelines and regulations and all experimental protocol was approved by the Institutional Review Board (IRB) (IRB # 201204036; Title: “Mapping the Human Connectome: Structure, Function, and Heritability”), and written informed consent was obtained from all participants. Our data analysis was performed in accordance with ethical guidelines of the Hanyang University ethics committee. The resting-state fMRI data was preprocessed with independent component analysis (ICA) with a new FSL tool FIX (FMRIB’s ICA-based X-noiseifier) (Salimi-Khorshidi et al., 2014). The “ICA-FIX cleaned” data included steps of co-registration, normalization, head motion correction, artifact rejection, and high-pass filtering (Glasser et al., 2013; Smith et al., 2013). The “ICA-FIX cleaned” resting-state fMRI data was used for identifying individuals.

Because the resting-state fMRI of the HCP consists of 1,200 volumes and each volume consists of approximately 220,000 voxels, functional ROIs were selected to reduce the spatial dimension of the data (Richiardi et al., 2015). Four hundred ninety nine functional ROIs were generated by subdividing 14 ICNs of 90 functionally defined ROIs from Shirer et al. (2012) and remaining cortical and subcortical regions through Ward clustering. Of 499 functional ROIs, 141 ROIs significantly overlapped with the 90 functional ROIs atlas and were used in further analysis. The BOLD signals were averaged for each ROI and converted into 141 × 1,200 two-dimensional (2D) arrays. The 2D-array fMRI data for each individual was normalized by subtracting the mean value and dividing by the SD. The initial 100 volumes of 1,200 volumes were used for learning of individual identification to overcome the limitation of using full length of fMRI time series as did Finn et al. (2015). Finally, a 141 × 100 2D array was used for each individual, and a total of 813 subject datasets were separated into 613 training datasets, 100 validation datasets, and 100 test datasets per eightfold cross-validation. The data used in this study were constructed as shown in Table 1.

**TABLE 1:** Subject scheme per eightfold cross-validation.

	**Subjects with both REST1 and REST2 present**
Training dataset	*n* = 613
Validation dataset	*n* = 100
Test dataset	*n* = 100
Total dataset	*n* = 813

*REST1 indicates resting-state fMRI acquired on day 1 and REST2 resting-state fMRI acquired on day 2.*

### Model

**FIGURE 1:**
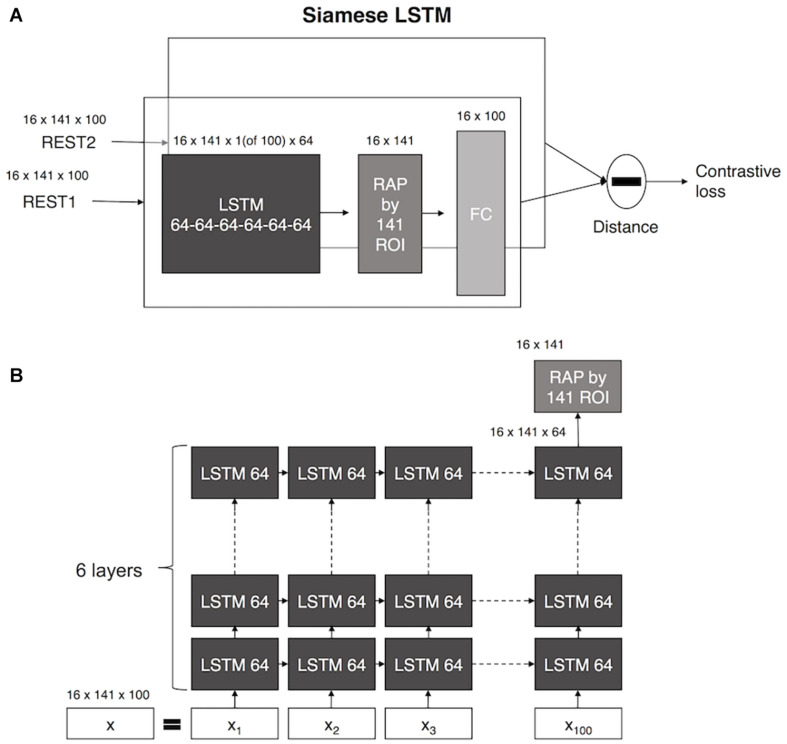
The architecture of Siamese LSTM. **(A)** A schematic diagram of Siamese LSTM model. **(B)** Detailed description of Siamese LSTM with 141 × 100 input 2D array.

Siamese LSTM consists of two identical LSTMs that share weights, as shown in Figure 1. The LSTM consists of six layers of 64 feature sizes, and the averaged fMRI data for each ROI at 100 time points was used as input. The RAP layer averaged 64 temporal features of each ROI extracted through LSTM, and it was added before a fully connected layer created a 100-dimensional latent space. The distance between pairs of fMRI data features in 100 dimensions was calculated, and the contrastive loss was used to train the Siamese LSTM with RAP by minimizing the distance if the two datasets are identical and maximizing the distance if the two datasets are non-identical.

#### Siamese LSTM With RAP

Long short-term memory consists of hidden state *h*_*(t)*_ for short-term memory and cell state *c*_*(t)*_ for long-term memory and three controllers for adjusting cell state: input gate *i*_*(t)*_, forget gate *f*_*(t)*_, and output gate *o*_*(t)*_. The input gate *i*_*(t)*_ adjusts the information of the input layer *x_(t)_* and the past hidden state *h*_*(t–1)*_ to be added to the cell state *c*_*(t)*_. The forget gate *f*_(*t*)_adjusts the information of past cell state *c*_*(t–1)*_. The output gate *o*_*(t)*_ adjusts the information of cell state *c*_*(t)*_ to be output to the hidden state *h*_*(t)*_ and the latest output layer *y*_*(t)*_. Because the gates *i*_*(t)*_, *f*_*(t)*_, and *o*_*(t)*_ use sigmoid functions, the output range is between 0 and 1. The equation of each unit of LSTM was defined as

(1) $$i_{\left(t\right)}=\sigma \,\left(W_{x\,i}^{T}\cdot x_{\left(t\right)}+W_{h\,i}^{T}\cdot h_{\left(t-1\right)}+b_{i}\right)$$

(2) $$f_{\left(t\right)}=\sigma \,\left(W_{x\,f}^{T}\cdot x_{\left(t\right)}+W_{h\,f}^{T}\cdot h_{\left(t-1\right)}+b_{f}\right)$$

(3) $$o_{\left(t\right)}=\sigma \,\left(W_{x\,o}^{T}\cdot x_{\left(t\right)}+W_{h\,o}^{T}\cdot h_{\left(t-1\right)}+b_{o}\right)$$

(4) $$c_{\left(t\right)}=f_{\left(t\right)}⊗c_{\left(t-1\right)}+i_{\left(t\right)}⊗\text{t}anh(W_{x\,c}^{T}\cdot x_{\left(t\right)}+W_{h\,c}^{T}\cdot h_{\left(t-1\right)}+b_{c})$$

(5) $$y_{\left(t\right)}=h_{\left(t\right)}=o_{\left(t\right)}⊗\text{t}\,a\,n\,h\,\left(c_{\left(t\right)}\right)$$

where *W*_*xi*_,*W*_*xf*_,*W*_*xo*_,*W*_*xc*_ are the weight matrices of the four layers each connected to the input vector *x*_*t*_, *W*_*hi*_,*W*_*hf*_,*W*_*ho*_,*W*_*hc*_ are the weight matrices of the four layers each connected to the past hidden state *h*_(*t–1*)_, *b*_*i*_,*b*_*f*_,*b*_*o*_,*b*_*c*_ are the bias in the four layers, and ⊗ is the element-wise product that controls each element of the cell state *c*_*(t)*_.

To extract temporal features for each of the 141 functional ROIs from a 141 × 100 input array, a 1 × 100 input vector was applied to LSTM. After applying LSTM, only the last time output layer of the temporal dynamic features learned for each ROI was passed to the next RAP layer. The RAP layer averaged temporal dynamic features extracted by LSTM for each ROI to maintain independence. This structure helped to visualize which ROIs had a significant impact on identification, after which a fully connected layer was mapped of the original data into a latent space of 100 dimensions by combining the temporal dynamic features of 141 ROIs.

#### Contrastive Loss

The contrastive loss is an objective function commonly used for contrastive training (Hadsell et al., 2006). Given one pair and a label, the distance between the pair with the positive label was minimized and the distance between a pair with a negative label maximized for being smaller than the margin parameter α. In this study, the margin parameter α was used as one. The distance of identical pairs converged to zero and the distance of non-identical pairs converged to one. The equation of contrastive loss was defined as

(6) $$J=\frac{1}{n}\,\sum_{i,j}^{n/2}{\text{y}}_{i,j}\,{\text{D}}_{i,j}^{2}+\left(1-{\text{y}}_{i,j}\right)\,{\left[\alpha -{\text{D}}_{i,j}\right]}_{+}^{2}$$

where *n* is the number of the batch, *D*_*i*,*j*_ = ∥*f*(*x*_*i*_)−*f*(*x*_*j*_)∥_2_, *f*(⋅) is the feature vector of latent space of the Siamese LSTM with RAP, *y*_*i*,*j*_ ∈ {0,1} is the label indicating whether a pair (*x*_*i*_,*x*_*j*_) is from the same class or not, and [⋅]_+_ is the maximize function max(0,⋅).

#### Implementation Details

**FIGURE 2:**
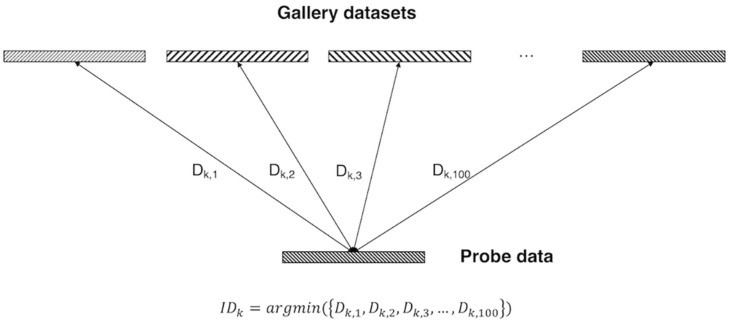
Identification analysis. Gallery datasets and probe data were represented by the latent vector of fully connected layers in 100 dimensions. Among the distance between the probe data and all gallery datasets, the gallery data of the minimum distance from the probe data was selected as the identification data.

In the training dataset, the pairs were combined as follows: subject “k” of REST1_RL and subject “k” of REST2_RL pairs were given a positive label, and subject “k” of REST1_RL and subject “k + 1” of REST2_RL pairs were given a negative label. The mini-batch size of one iteration was 32, so when the weight was updated, it learned with 16 labels and 16 pairs (eight positive pairs and eight negative pairs). In the validation and test dataset, the pairs were combined as follows: 100 pairs were created with subject “k” of REST1_RL and all subjects of REST2_RL, and a total 10,000 pairs (100 positive pairs, 9,900 negative pairs) were generated from validation and test datasets. To sum up, the distance between one probe data (i.e., subject k of REST1_RL) and 100 gallery data (i.e., all subjects of REST2_RL) was calculated and the gallery with the minimum distance selected. Individual identification performance was evaluated by confirming that the selected gallery was the same data as the probe data {i.e., *ID*_*k*_ = *argmin*[(*D*_*k*,1_,*D*_*k*,2_,*D*_*k*,3_,*D*_*k*,100_)]} (Figure 2).

Dropout ratio was set to 0.2 within the input layer of LSTM. The L2 regularization was set to 0.1. Adam optimizer (Kingma and Ba, 2014) (β_1_ = 0.9,β_2_ = 0.999)was used with the initial learning rate 1e-5. A dropout was not used in the fully connected layer. Non-linear function of the fully connected layer was used as a hyper tangent function, with the output range of −1 to 1. To prevent overfitting, early stopping-based validation accuracy was used, and then the test dataset was evaluated. The feature size of LSTM layers was selected as “64-64-64-64-64-64” of six layers. The model training was performed with a randomly shuffled training dataset.

### Visualization of Features for Individual Identification

The RAP layer was observed to understand the learning of Siamese LSTM for individual identification. The features of the RAP layer represent temporal dynamic features in ROIs of 14 ICNs. A weight matrix of 141 × 100 in the fully connected layer was also observed to determine which features of ROI are mainly used to create a 100-dimensional latent vector. Z-score of normal distribution was calculated to visualize which ROI is significant for individual identification, and z-values greater than 1 are distributed in the top 30%. One hundred weights for each ROI were averaged and standardized by the mean and SD of 141 ROIs. The ROIs with standard values greater than 1 were determined as important for individual identification.

Furthermore, the occlusion method was used for visualization of important ROIs for individual identification (Zeiler and Fergus, 2014). BOLD signals of a specific ROI were zeroed, and the occluded BOLD signals used as input data of Siamese LSTM with RAP. When each ROI was zeroed, the decreased performance was determined as the importance of each ROI. For instance, if a subject cannot be identified when BOLD signals of a specific ROI were zeroed, the importance value of the corresponding ROI was increased by 1.

## Results

### Performance of Siamese LSTM With RAP for Individual Identification

Identification accuracy of the suggested model was calculated by enumerating the correct *ID*_*k*_ of validation and test datasets consisting of 100 subjects each. Table 2 shows the accuracy of eightfold cross-validation. The average accuracy of the eightfold cross-validation was 97.88% for the test dataset. Table 3 is a comparison of the test accuracy of other studies using 100 time points of fMRI. The traditional machine learning (Finn’s method) was also tested on the number of subjects and volumes (Supplementary Table 1). The Siamese LSTM with RAP used half the parameters of the previous highest performing model (Wang et al., 2019), and yet the performance was comparable. The advantage of fewer parameters is that the model can run with a relatively small memory GPU or CPU.

**TABLE 2:** Accuracy of eightfold cross-validation for individual identification.

**Model**	**Onefold Acc (valid/test)**	**Twofold Acc (valid/test)**	**Threefold Acc (valid/test)**	**Fourfold Acc (valid/test)**	**Fivefold Acc (valid/test)**	**Sixfold Acc (valid/test)**	**Sevenfold Acc (valid/test)**	**Eightfold Acc (valid/test)**
Siamese LSTM (64-64-64-64-64-64) + RAP	0.97/1.0	0.99/0.96	0.99/0.97	0.99/0.99	0.98/0.95	1.0/0.99	0.98/1.0	1.0/0.97

*The best performance was achieved using the Siamese LSTM 64-64-64-64-64-64.*

*The average accuracies of the eightfold cross-validation were 0.9875 ± 0.010 for the validation dataset and 0.9788 ± 0.018 for the test dataset.*

**TABLE 3:** Identification performance according to the model.

**Model**	**Input data**	**Number of trainable parameters/Feature extraction**	**Test accuracy using 100 time points**
Finn et al. (2015)	FC	.	0.7
Chen and Hu (2018)	BOLD signal	405K/380K	0.9443
Wang et al. (2019)	BOLD signal	382K/3.8K	0.9850
Siamese LSTM + RAP	BOLD signal	196K/0.14K	**0.9788 ± 0.018**

*The average accuracy for the eightfold cross-validation test dataset is displayed in bold.*

### Identification Performance According to Siamese LSTM Structure

The performance according to layer width of LSTM was compared (Table 4). For each layer of the Siamese LSTM with RAP, the performance of the fixed layer width at 64 was slightly better than that of the layer width which doubled per layer in all cases. The performance depending on the depth of LSTM was compared (Table 5). When stacking layers from 3 to 9 with the fixed layer width at 64, the best performance was obtained when six layers were stacked. Furthermore, the performance according to the RAP layer of Siamese LSTM with RAP was compared (Table 6). It was better to add the RAP layer than to connect the fully connected layer directly after the LSTM structure. The RAP layer for ease of visualization not only reduced the number of parameters (1,084 K without RAP vs. 196 K with RAP) but also improved the identification performance.

**TABLE 4:** Evaluation of effect of layer width of LSTM on identification performance.

**Model**	**Layer depth**	**Layer width**	**Avg-fold accuracy (valid/test)**
Siamese LSTM + RAP	Four layers	8-16-32-64	0.9838 ± 0.019/0.9725 ± 0.0183
		64-64-64-64	0.9888 ± 0.012/0.9750 ± 0.013
	Five layers	8-16-32-64-128	0.9662 ± 0.035/0.9550 ± 0.043
		64-64-64-64-64	0.9900 ± 0.009/0.9725 ± 0.021
	Six layers	8-16-32-64-128-256	0.9788 ± 0.021/0.9637 ± 0.021
		64-64-64-64-64-64	0.9875 ± 0.010/**0.9788** ± **0.018**

*The best average accuracy for the eightfold cross-validation is displayed in bold.*

**TABLE 5:** Evaluation of effect of layer depth of LSTM on identification performance.

**Model**	**Layer depth**	**Avg-fold accuracy (valid/test)**
Siamese LSTM + RAP	Three layers (64-64-64)	0.9775 ± 0.030/0.9550 ± 0.020
	Four layers (64-64-64-64)	0.9888 ± 0.012/0.9750 ± 0.013
	Five layers (64-64-64-64-64)	0.9900 ± 0.009/0.9725 ± 0.021
	Six layers (64-64-64-64-64-64)	0.9875 ± 0.010/**0.9788** ± **0.018**
	Seven layers (64-64-64-64-64-64-64)	0.9925 ± 0.009/0.9700 ± 0.022
	Eight layers (64-64-64-64-64-64-64-64)	0.9887 ± 0.011/0.9750 ± 0.020
	Nine layers (64-64-64-64-64-64-64-64-64)	0.9875 ± 0.016/0.9688 ± 0.016

*The best average accuracy for the eightfold cross-validation is displayed in bold.*

**TABLE 6:** Evaluation of effect of RAP layer on identification performance.

**Model**	**Avg-fold accuracy (valid/test)**
Siamese LSTM + No RAP	0.9838 ± 0.013/0.9387 ± 0.028
Siamese LSTM + RAP	0.9875 ± 0.010/**0.9788** ± **0.018**

*The Siamese LSTM model consists of 64-64-64-64-64-64, which showed the highest accuracy.*

*The best average accuracy for the eightfold cross-validation is displayed in bold.*

### Spatial Features for Individual Identification

**FIGURE 3:**
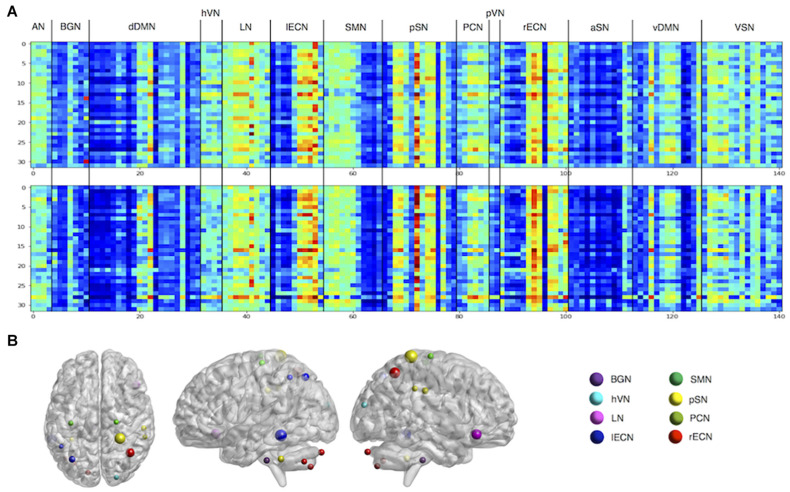
Visualization of features for individual identification. **(A)** The features of the RAP layer. Each matrix represents RAP features extracted from two of the eight cross-validation models. The rows are each mini-batch, and the columns the ROI. **(B)** The z-scored weights of fully connected layers. ROIs with z-values greater than 1 are plotted. AN, auditory network; BGN, basal ganglia network; dDMN, dorsal default mode network; hVN, higher visual network; LN, language network; lECN, left executive control network; SMN, sensorimotor network; pSN, posterior salience network; PCN, precuneus network; pVN, primary VN; rECN, right ECN; aSN, anterior SN; vDMN, ventral DMN; VSN, visuospatial network.

The visualization method was used to observe the weights learned by Siamese LSTM with RAP (Figure 3). Through the extracted features of the RAP layer, we were able to determine which ROI feature was used by Siamese LSTM with RAP for individual identification. We further examined which ROI was significant to create a 100-dimension latent vector by standardizing the 141 × 101 weight matrix of the fully connected layer (Figure 3B). The ROI features of language network (LN), left executive control network (lECN), posterior salience network (pSN), and right ECN (rECN) contributed the most in the RAP and fully connected layer, especially the parietal regions of the brain. The right postcentral gyrus of pSN, the left middle temporal gyrus of lECN, the right superior parietal lobule of rECN, the right inferior frontal gyrus of LN, and the left superior parietal lobule of lECN (z-values: 4.917, 4.428, 3.782, 3.450, and 2.290, respectively) ranked the highest among the important ROIs for individual identification.

**FIGURE 4:**
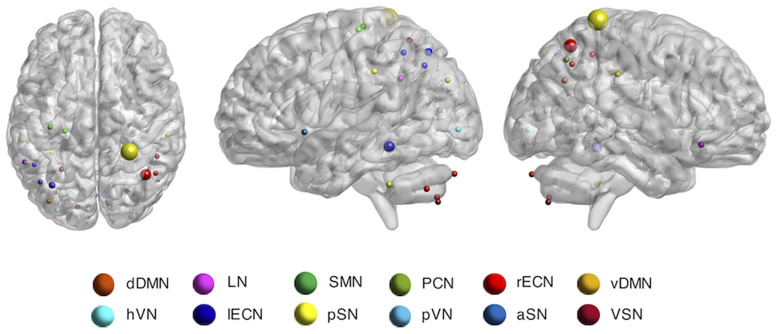
The importance value of ROI for individual identification by using occlusion method. dDMN, dorsal default mode network; hVN, higher visual network; LN, language network; lECN, left executive control network; SMN, sensorimotor network; pSN, posterior salience network; PCN, precuneus network; pVN, primary VN; rECN, right ECN; aSN, anterior SN; vDMN, ventral DMN; VSN, visuospatial network.

Furthermore, the occlusion method was used to visualize the significant ROI for individual identification (Figure 4). For all test datasets, the importance value of the ROI was increased by one if the identification was not performed when each ROI was excluded. The importance of ROIs measured by the occlusion method overlapped with the ROIs determined by the z-score weight of the fully connected layer (Figure 3B), and the parietal regions still contributed greatly to individual identification. The ICN-based occlusion method was also performed. For all test datasets, the importance value of that ICN was increased by one if the identification was not performed when each ICN was excluded (Table 7). The result also showed that lECN, pSN, and rECN were more important for individual identification.

**TABLE 7:** Important value of ICN by occlusion method.

**ICN**	**Important value**
AN	0
BGN	0
dDMN	8
hVN	0
LN	8
lECN	121
SMN	0
pSN	155
PCN	0
pVN	1
RECN	95
aSN	1
vDMN	3
VSN	1

*AN, auditory network; BGN, basal ganglia network; dDMN, dorsal default mode network; hVN, higher visual network; LN, language network; lECN, left executive control network; SMN, sensorimotor network; pSN, posterior salience network; PCN, precuneus network; pVN, primary VN; rECN, right ECN; aSN, anterior SN; vDMN, ventral DMN; VSN, visuospatial network.*

### Latent Space Visualization by t-SNE

**FIGURE 5:**
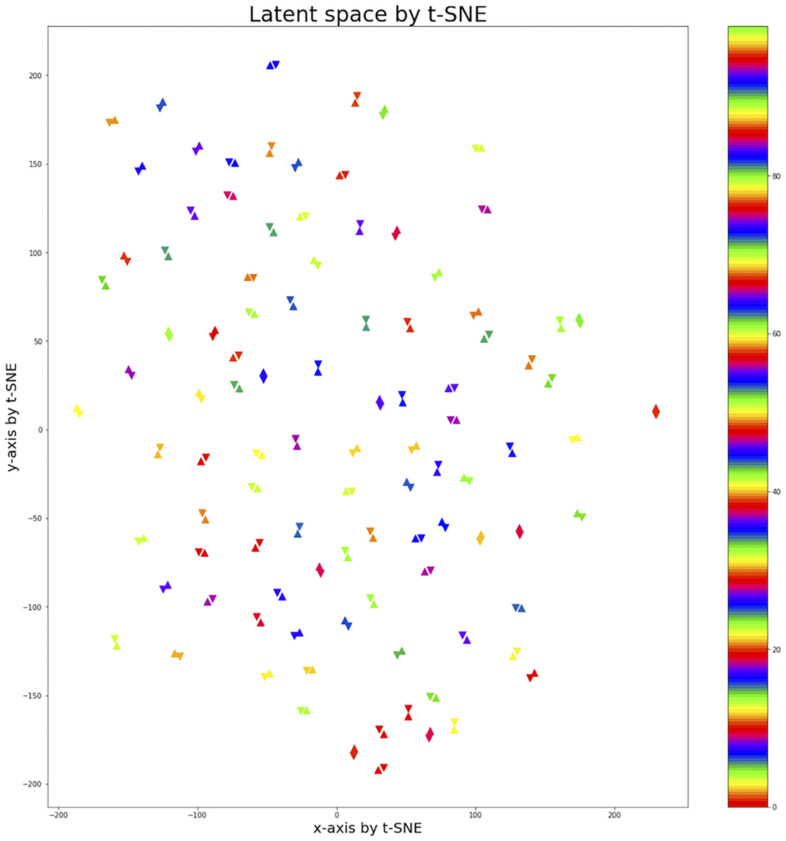
Latent space of 100 subject pairs by using t-SNE. A hundred subject pairs are expressed in the 2-dimensional space using the t-SNE method to observe how the subject pairs are located in the 100-dimensional latent space created by fully connected layer. The uptight triangle represents REST1 data and the upside-down triangle represents REST2 data. The colors indicate pairs of different subjects. The identical pairs were located closest to the latent space.

To observe the subject pair data mapped into the 100-dimensional latent space by the fully connected layer, t-Distributed Stochastic Neighbor Embedding (t-SNE) (Maaten and Hinton, 2008) was used to represent 100 pairs of data on the 100-dimensional latent space in 2D space. An upright triangle was used to represent REST1 data and an upside-down triangle REST2 data (Figure 5). Using the contrastive loss to map identical pairs into the nearest location, the distance of identical data pair was close to zero, and the distance of non-identical data pair was close to one. The result showed that the identical pairs are nearest to each other in latent space.

## Discussion

In this study, the Siamese LSTM with RAP was implemented for individual identification using initial 100 volumes of fMRI data. Siamese LSTM, where two identical LSTM share weights, was a suitable model for extracting temporal dynamic features from a pair of dynamic sequence data. Contrastive loss calculated the distance between feature vectors extracted by the Siamese LSTM with RAP and minimized the distance of positive pairs and maximized the distance of negative pairs. Performance of the Siamese LSTM with RAP was more robust for newly added datasets. For the test dataset, the identical pairs were mapped into the nearest location in 100-dimensional latent space. Furthermore, the features learned by the Siamese LSTM with RAP were observed by the visualization method, and the key ICNs for individual identification were identified.

### Robust Siamese LSTM With RAP in New Dataset

The Siamese LSTM with RAP achieved an average of 97.88% individual identification accuracy for the test dataset with eightfold cross-validation (Table 2). Individual identification accuracy using BOLD signal is higher than that using FC (Table 3) because RNN-based models use the dynamic features of BOLD signal.

Wang et al. (2019) achieved an individual identification accuracy of 98.50% using a short-time BOLD signal. However, because their model classifies 100 subject labels identical to the training data using the Softmax as the last layer, it is unclear whether the same performance will be achieved for new training with additional subjects. On the other hand, the Siamese LSTM with RAP guarantees robust performance of individual identification accuracy even for newly added subjects because our model learned to minimize the distance of positive pairs and to maximize the distance of negative pairs. In addition, the Siamese LSTM with RAP improved the reliability of the individual identification performance by validating the performance of 800 test datasets using the eightfold cross-validation method compared with other studies conducted with only 100 subjects. Moreover, the Siamese LSTM with RAP is a more efficient model because it performs consistently well despite a small number of learnable parameters.

### Siamese LSTM Structure Related to Performance Improvement

The fixed width of the LSTM for each layer achieved better accuracy than the widening width, and the deeper model did not enhance accuracy (Tables 4, 5). To improve learning performance, appropriate width and depth of the model should be selected depending on the data, which seems to be related to the complexity of the training data.

The RAP structure of the Siamese LSTM with RAP played a key role in improving the accuracy of individual identification. When the RAP structure was not used, the average accuracy of the eightfold cross-validation was 93.87% (Table 6). Models using RAP structures were developed by taking into account ROI-specific dynamic features, thus preventing overfitting of the training dataset and improving individual identification accuracy (Supplementary Figure 1). When using the same hyperparameter, the model without RAP has reduced accuracy on validation dataset after 50 epochs, whereas the model with RAP has reduced accuracy on validation dataset after 300 epochs. Although the model without RAP can quickly improve the individual identification performance of the training data with more trainable parameters, it does not guarantee regularization for validation and test datasets. Considering the dynamic feature of each ROI helped to assess the importance of each ROI in individual identification as well as to regularize the model to the data.

### Spatial Features Learned by Siamese LSTM With RAP and Consistency With Previous Findings

The lECN, rECN, pSN, and LN have been identified as key ICNs for individual identification by occlusion method and visualization of the RAP and fully connected layers (Figures 3, 4 and Table 7). Among the ROIs of the lECN and rECN, which consist of frontal, parietal, and middle temporal regions, only the ROIs in the parietal and middle temporal regions substantially contributed to individual identification. The ECN is also known to have a significant effect on individual identification in other studies (Finn et al., 2015; Liu et al., 2018; Wang et al., 2019). Frontal, parietal, and middle temporal cortex belonging to the ECN are the regions where cortical folding occurs more than in other brain regions (Zilles et al., 1988), and frontal, parietal, and middle temporal gyrus are known to have high sulcal depth variability due to evolutionary expansion of the cortical surface (Hill et al., 2010). Furthermore, the evolutionary cortical surface expansion and inter-subject variability of FC are highly correlated in the brain, especially in the regions of the ECN with a high inter-subject variability (Mueller et al., 2013). Based on these, the ECN consisting of frontal, parietal, and middle temporal regions is likely to affect individual identification due to structural differences among individual brains. Posterior SN located in parietal regions of the brain was also identified for its significance in individual identification. The results implied that the dynamic features of BOLD signal in the described regions may have a high inter-subject variability. Furthermore, for the LN, brain activation in right inferior frontal gyrus reported positive correlations with individual reading span scores in studies of adults through the reading and listening comprehension task (Buchweitz et al., 2009) and with individual syntactic comprehension scores in studies of children through the sentence-verification task (Yeatman et al., 2010). From these results, it was found that the spatial features learned by Siamese LSTM with RAP were already reported as regions with high inter-subject variability.

Previous fMRI studies have demonstrated that time factors affect cognitive performance in working memory, attention, and executive function within a day (Anderson and Revelle, 1994; May et al., 2005; Schmidt et al., 2007; Gorfine and Zisapel, 2009). Despite that, the spatial pattern of ICN has been proven to be consistent across multiple sessions, days, and subjects, and many studies have assumed long-term stable-spontaneous ICNs (Damoiseaux et al., 2006; Chen et al., 2008; Meindl et al., 2010). However, the variability of individual ICNs over time has been shown to vary with the ICN sub-network, reminding us of the importance of the temporal dynamics of resting-state fMRI (Park et al., 2012). In particular, the right and left fronto-parietal networks, superior parietal network, and secondary motor network showed high intra-class correlation, consistent with the results of our study emphasizing the parietal regions for identifying individuals. Although this study trained the Siamese LSTM with RAP to identify fMRI data of one person scanned in 1 day as fMRI data of the same person in the consecutive day, it would also be a good approach to learn the model to identify individual uniqueness that remains unchanged after 1 or 2 years. In a longitudinal resting-state fMRI study, which scans were done weekly over 3.5 years, the reproducibility of the spatial map of 14 ICNs was divergent. Furthermore, ICNs with a low reproducibility in the spatial map showed a low reproducibility in the temporal fluctuation magnitude. Among the ICNs, rECN, and lECN presented a high reproducibility for spatial map and temporal signal fluctuation magnitudes, which was consistent with the results of this study (Choe et al., 2015). Consistently with the previous longitudinal studies, the Siamese LSTM with RAP is expected to perform well enough on the longitudinal dataset. In addition, the key ICNs for individual identification on the daily scan dataset are expected to be key ICNs for individual identification on the longitudinal dataset.

In this study, individual identification was performed using Siamese LSTM with RAP, but it is still insufficient to use resting-state fMRI as a real fingerprint because learning and evaluation were performed with the dataset acquired at daily intervals. To be used as real fingerprints, the learning and evaluation process should be conducted with dataset acquired monthly or annually. The Siamese LSTM with RAP was trained to extract dynamic temporal features, but did not show temporal features. It is necessary to study the meaning of important temporal features as well as showing important temporal features in future studies. In conclusion, we combined LSTM and Siamese network to achieve robust individual identification performance without additional learning on newly added datasets. Furthermore, we applied a novel RAP layer to obtain key ICNs for individual identification and improvement in individual identification performance. The high-level performance of individual identification through the Siamese LSTM with RAP proved that the dynamic resting-state fMRI is unique enough to perform for individual identification. The spatial features for individual identification were mainly identified in the parietal regions, which are consistent with previous findings of high inter-subject variability in the parietal regions. These results are expected to be a good foundation for future individual characteristic research.

## Data Availability Statement

Publicly available datasets were analyzed in this study. This data can be found here: https://www.humanconnectome.org/.

## Ethics Statement

The experiments were performed in accordance with relevant guidelines and regulations and all experimental protocol was approved by the Institutional Review Board (IRB) (IRB #201204036; Title: “Mapping the Human Connectome: Structure, Function, and Heritability”), and written informed consent was obtained from all participants.

## Author Contributions

Y-HP analyzed the data and wrote the article. SS wrote the article. SK analyzed the data. J-ML designed the experiments and revised the manuscript. All authors contributed to the article and approved the submitted version.

## Conflict of Interest

The authors declare that the research was conducted in the absence of any commercial or financial relationships that could be construed as a potential conflict of interest.

## Footnotes

1 https://www.humanconnectome.org/
