# Supplementary material for: Key Intrinsic Connectivity Networks for Individual Identification With Siamese Long Short-Term Memory
Source: Front Neurosci. 2021 Jun 18;15:660187. doi: 10.3389/fnins.2021.660187 (PMC8249867; doi:10.3389/fnins.2021.660187)
Supplement: Supplementary file 1 [file Table_1.DOCX]

Supplementary Material

# Supplementary Figure and Table

## Supplementary Figure


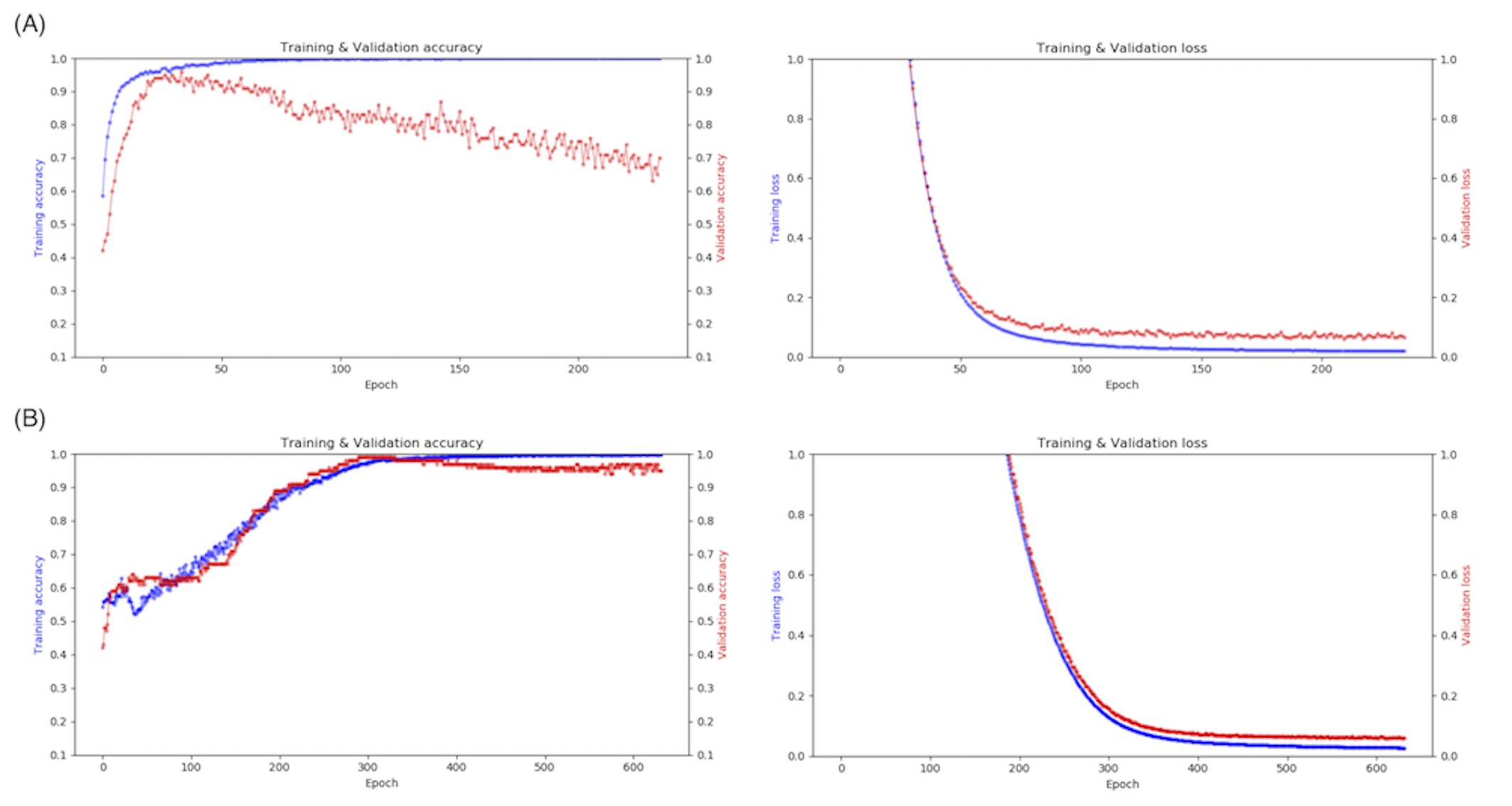


**Supplementary Figure 1.** **Learning difference in training and validation dataset with or without RAP layer** (A) Accuracy and loss functions of Siamese LSTM not using RAP structure along epochs. (B) Accuaracy and loss functions of Siamese LSTM using RAP structure along epochs.

## Supplementary Table

**Supplementary Table 1. Identification performance according to the number of subjects and volumes**

|  | **Q2 release**  **(Finn’s paper)** | **N=300** | **N=500** | **N=700** | **N=813(This paper)** |
| --- | --- | --- | --- | --- | --- |
| Volume = 1200 | 0.792 (0.727, 0.856) | 0.680 (0.620, 0.740) | 0.684 (0.614, 0.754) | 0.667 (0.588, 0.745) | 0.656 (0.579, 0.733) |
| Volume = 120 | 0.499 (0.545, 0.454) | 0.377 (0.400, 0.353) | 0.383 (0.392, 0.374) | 0.357 (0.357, 0.357) | 0.348 (0.345, 0.351) |

*The identification performance indicates that ‘average accuracy (accuracy if source is REST1, accuracy if source is REST2)’.
